# Supplementary material for: High-Level Production of Bacteriotoxic Phospholipase A1 in Bacterial Host Pseudomonas fluorescens via ABC Transporter-Mediated Secretion and Inducible Expression
Source: Microorganisms. 2020 Feb 11;8(2):239. doi: 10.3390/microorganisms8020239 (PMC7074900; doi:10.3390/microorganisms8020239)
Supplement: Supplementary file 1 [file microorganisms-08-00239-s001.pdf]

High-Level Production of Bacteriotoxic Phospholipase A1 in Bacterial host *Pseudomonas fluorescens* Via ABC-Transporter Mediated Secretion and Inducible xpression

Jiyeon Park [1,2,†], Gyeong Tae Eom [3,†], Joon Young Oh [4], Ji Hyun Park [4], Sun Chang Kim [2,5], Jae Kwang Song [4], Jung Hoon Ahn [1,5,*]

[1]Korea Science Academy of Korea Advanced Institute of Science and Technology, Busan 47162, Korea; jyp131@kaist.ac.kr
[2]Intelligent Synthetic Biology Center, 291 Daehak-ro, Yuseong-gu, Daejeon 305-701, Korea; sunkim@kaist.ac.kr
[3]Research Center for Bio-based Chemistry, Korea Research Institute of Chemical Technology (KRICT) 1, Ulsan, 44429, Korea; eomgt@krict.re.kr
[4]Research Center for Bio-based Chemistry, Korea Research Institute of Chemical Technology (KRICT), Daejeon 34114, Korea; jyoh@krict.re.kr (J.Y.O.); jhpark2@krict.re.kr (J.H.P.); ajee0414@gmail.com (J.K.S.)
[5]Department of Biological Sciences, Korea Advanced Institute of Science and Technology, Daejeon 34141, Korea
*Correspondence: hoony@kaist.ac.kr (J.H.A.); Tel.: 8251-606-2335
†These authors contributed equally to this work

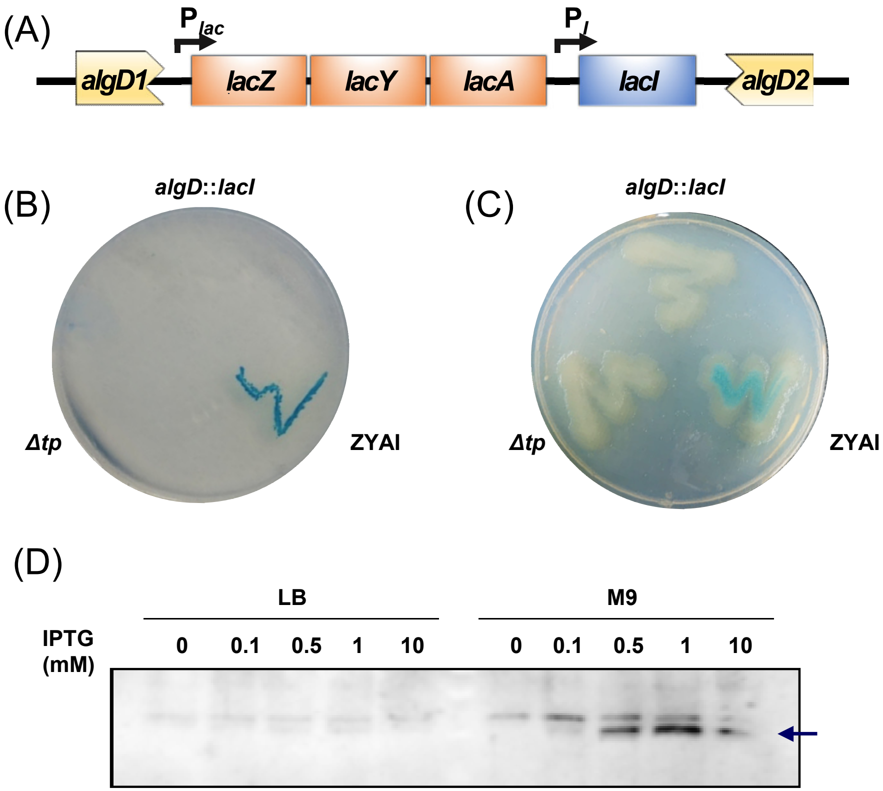

Figure S1. Construction of *P. fluorescens* ZYAI. **(A)** Genomic organization of knocked-in *P. fluorescence* ZYAI. **(B)** Each recombinant *P. fluorescens* was streaked on an M9 agar plate containing 40 µg/ml X-Gal, 1 mM IPTG, and 0.6 % lactose as sole carbon source. *P. fluorescens Δtp* and *algD::lacI* could not grow in lactose supplemented M9 medium. **(C)** It was streaked similarly on an M9 agar plate containing 40 µg/ml X-Gal, 1 mM IPTG, 0.6 % lactose, and 2 % glucose. Only *P. fluorescens* ZYAI could hydrolyze X-gal to show a blue colony. **(D)** Western blot for the analysis of secreted PlaA from *P. fluorescens* ZYAI in the different IPTG concentrations on LB or M9 medium. The arrow indicates PlaA (45 kDa). *Δtp*: *ΔtliA ΔprtA, algD*::*lacI*: *lacI* knocked-in *Δtp, ZYAI: lacZYA* and *lacI* knocked in *Δtp.*

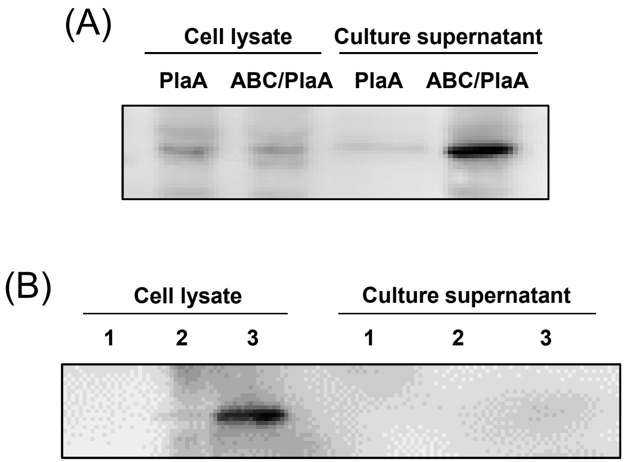

Figure S2. Secretion of PlaA by the ABC transporter system. **(A)** *P. fluorescens* cells harboring the PlaA-encoding gene fused with LARD3 were cultured at 25 °C for 4 days in the presence or absence of the ABC transporter. Western blot analysis was performed in the cell and in the supernatant. **(B)** PlaA expression without LARD3 was analyzed. Three plasmids were used for this purpose: pDSK (1), pDSK-PlaA (2), and pDSK-PlaA/PlaS (3), with control, PlaA, and PlaA/PlaS in pDSK519, respectively. Culture conditions and analyses used were the same as above.
